# Sex differences in treatment patterns for non-advanced muscle-invasive bladder cancer: a descriptive analysis of 3484 patients of the Netherlands Cancer Registry

**DOI:** 10.1007/s00345-022-04080-6

**Published:** 2022-07-01

**Authors:** Anke Richters, Anna M. Leliveld, Catharina A. Goossens-Laan, Katja K. H. Aben, Berna C. Özdemir

**Affiliations:** 1 Department of Research and Development, The Netherlands Comprehensive Cancer Organisation, Utrecht, The Netherlands; 2 Radboud University Medical Center, Radboud Institute for Health Sciences, Nijmegen, The Netherlands; 3 Department of Urology, University Medical Center Groningen, Groningen, the Netherlands; 4 Department of Urology, Alrijne Hospital, Leiderdorp, the Netherlands; 5 Department of Oncology, Bern University Hospital and University of Bern, Bern, Switzerland

**Keywords:** Sex differences, Gender, Bladder cancer, MIBC, Urothelial carcinoma, Treatment pattern, Treatment allocation, Cancer registry

## Abstract

**Purpose:**

Bladder cancer (BC) is a common malignancy with well-established differences in incidence, clinical manifestation and outcomes between men and women. It is unknown to what extent disparities in outcomes are influenced by differences in treatment approaches. This paper describes treatment patterns among men and women with muscle-invasive BC focusing on curative treatment (radical cystectomy or trimodal therapy).

**Methods:**

A retrospective population-based cohort study was performed with data from the Netherlands Cancer Registry. All patients newly diagnosed with muscle-invasive, non-advanced BC (MIBC, cT2-4a, N0/X, M0/X) in the years 2018, 2019 and 2020 were identified. Patient and tumor characteristics and initial treatment were compared between men and women with descriptive statistics and multivariable logistic regression analyses.

**Results:**

A total of 3484 patients were diagnosed with non-advanced MIBC in 2018–2020 in the Netherlands, of whom 28% were women. Women had higher T-stage and more often non-urothelial histology. Among all strata of clinical T-stage, women less often received treatment with curative intent (radical cystectomy [RC] or trimodality treatment). Among RC-treated patients, women more often received neoadjuvant treatment (except for cT4a disease). After adjustment for pre-treatment factors, odds ratios were indicative of women having lower probability of receiving curative treatment and RC specifically, and higher probability to receive NAC when treated with RC then men, although not statistically significant.

**Conclusions:**

Considerable differences in treatment patterns between men and women with MIBC exist. A more considerate role of the patient’s sex in treatment decisions could help decrease these differences and might mitigate disparities in outcomes.

**Supplementary Information:**

The online version contains supplementary material available at 10.1007/s00345-022-04080-6.

## Introduction

Urothelial carcinoma of the bladder (BC) is a common cancer, ranking tenth in worldwide absolute cancer incidence. It is a particularly illustrative example of the differential impact of both sex and gender in a non-sex-related disease. Men are three to four times more often affected than women with a worldwide age standardized incidence rate per year of 9.6 per 100,000 for men and 2.4 per 100,000 for women [1]. This sex disparity in risk persists even after adjusting for differences in occupational hazards and smoking prevalence and intensity [2, 3], possibly due to biological differences. While cancer risk is higher in men, women present with more advanced disease at the time of diagnosis [4–6]. This is largely due to diagnostic delay, as women with hematuria are more likely to be diagnosed with a urinary tract infection and receive symptomatic treatment and less likely to undergo abdominal imaging [7, 8] and be referred for urological evaluation [8, 9].

Women have a higher cancer-specific mortality risk and lose a greater fraction of their life expectancy to BC [10]. Although women have worse survival rates even after adjusting for disease stage at diagnosis, this excess mortality is present only in the first 2 years after diagnosis, thereafter the mortality rate of women is lower than that of men [4, 6, 11, 12]. According to an analysis of the SEER database between 1990 and 2005, differences in prognostic factors such as age, tumor stage, grade and histological type account only for about 30% of the sex differences in BC mortality [11], suggesting that other factors such as differences in tumor biology, choice and efficacy of treatments and delay in treatment could contribute to this excess female mortality [12, 13].

There are only limited data on potential sex differences in treatment allocation and efficacy with conflicting results. Data from the SEER database from 1992 to 1999 do not show a significant difference in RC rates between men and women [14, 15]. A more contemporary cohort study from the Netherlands is indicative of lower probabilities of RC among women after adjustment for age, stage, socioeconomic status and type of hospital, although not significant [16]. Some single-center analyses have reported that women undergoing RC have longer operative time, more blood loss, and more frequent perioperative complications and a higher 90-day mortality risk. In addition, the rates of pelvic lymph node dissection and continent diversion are lower for women [17, 18]. In a recent analysis of a large US RC cohort, female sex was associated with longer operative time and length of stay, while reoperation, readmission and 30-day mortality rates were similar [19]. Other single-center or multicenter analyses, however, have not found significant sex differences in surgery complications, resection margins or lymph node count but consistently reported a poorer outcome for women [5, 20, 21].

To obtain insight into differences in treatment between male and female patients, we analyzed treatment patterns from a contemporary cohort of BC patients diagnosed in 2018–2020, with a focus on curative treatment, defined as radical cystectomy or trimodal therapy (TMT).

## Patients and methods

### Cohort

For this population-based cohort study, all patients newly diagnosed with muscle-invasive BC (MIBC) defined as cT2-4b, cN0/X, cM0/X (without previous invasive bladder cancer diagnosis, i.e., no earlier T1 diagnosis), diagnosed between 2018 and 2020 were identified from the Netherlands Cancer Registry (NCR). All histologies were included, except for urachus tumors.

This nationwide population-based registry, held by the Netherlands Comprehensive Cancer Organisation (IKNL), includes data on all cancer diagnoses for residents of the Netherlands since 1989.

The Privacy Review Board of the NCR approved the study and waived the need for written informed consent.

### Clinical data

Data from the NCR about patient and tumor characteristics, disease stage, initial treatment, comorbidities, performance status, laboratory test results, treatment details (e.g., number of cycles and dose adjustments) were used. These data have been collected by specialized data managers at the NCR directly from electronic health records in all hospitals in the Netherlands. Clinical data were completed approximately 9 months after diagnosis.

The clinical TNM stage was based on physical examination, imaging and transurethral resection of the tumor (TUR). Radical cystectomy with or without neoadjuvant treatment with platinum-based chemotherapy or immune checkpoint inhibitors, and trimodal therapy (maximal TUR with subsequent chemoradiation) were considered treatments with curative intent.

Performance status documented as the Karnofsky performance score (KPS) was converted to Eastern Cooperative Oncology Group (ECOG) score, as follows: KPS 100 = ECOG 0; KPS 80–90 = ECOG 1; KPS 60–70 = ECOG 2; KPS 40–50 = ECOG 3; and KPS 10–30 = ECOG 4 [9]. Age-adjusted Charlson Comorbidity Index (aCCI) was used, excluding the presence of MIBC itself (i.e., the morbidity of interest). Renal function was categorized as 0–39 (cisplatin ineligible), 40–59 (eligible for split-dose cisplatin), and 60–79 or ≥ 80 mL/min/1.73 m^2^ (cisplatin eligible). Hospital type was categorized into three groups. University hospitals are allied with major Dutch universities, with a specific focus on research and education in addition to patient care, and provide specialized care. Non-university teaching hospitals also work with the university hospitals to aid in the training of professionals, and offer some of the more specialized treatments. General hospitals provide high volumes of standard health care for less specialized issues and generally refer patients to more specialized facilities. In case, more hospitals were involved in the diagnosis and/or treatment, the hospital where the diagnostics were performed was included in the analyses.

### Statistical analysis

Data were summarized as frequencies with percentages, or mean values with standard deviation, or median values with interquartile range. Standardized differences between distributions among men and women patients were calculated as a measure of difference between both sexes [22], which are not dependent on sample size and are standardized to values between 0 (no difference) and 1 (maximum difference). This measure was considered appropriate given the descriptive nature and given that all results are based on a population-based cohort or subgroups thereof that were not specifically powered to test a priori defined hypotheses for statistical significance. There is no established manner to interpret the magnitude of standardized differences, as the clinical relevance depends on the context, but the authors considered all standardized differences above 0.1 to be relevant for this study. Multivariable logistic regression was performed to assess the association of sex with treatment patterns adjusted for other pre-treatment factors.

## Results

In the years 2018–2020, a total of 3484 patients were diagnosed with MIBC without nodal or distant metastasis, of whom 28% (*n* = 992) were women. Median age at diagnosis was 74 years for both sexes (Table 1). The performance status at diagnosis was 0 or 1 in 54% of men, and 47% of women, respectively. Most patients were eligible for cisplatin-based therapy based on only their renal function. The ineligible group consisted of 7% of the men and 8% of the women having an eGFR of 0–30 ml/min and 17% and 16% had a eGFR 30–50, respectively. Women were diagnosed with more advanced tumors, with cT2 in 64% and cT4a in 10% of the cases, while men more often presented with a cT2 tumor (72%) and only in 5% with a cT4a tumor (standardized difference: 0.19). Women also had tumors with non-urothelial histology more often (10 vs 6%, standardized difference: 0.22).

**Table 1:** Patient and disease characteristics of all muscle-invasive, non-advanced bladder cancer patients in the Netherlands diagnosed in 2018–2020, by sex

	Men		Women		Standardized differences
	*N*	%	*N*	%	
All	2492	72	992	28	
Age at diagnosis					
0–50	40	2	25	3	0.12
51–60	225	9	103	10	
61–70	623	25	244	25	
71–80	957	38	331	33	
> 80	647	26	289	29	
Median, IQR	74	67–81	74	66–82	
Age-adjusted CCI					
< 2	130	5	70	7	0.17
2–3	878	35	398	40	
4	570	23	198	20	
5	387	16	133	13	
> 5	425	17	148	15	
Unknown	102	4	45	5	
Performance status at diagnosis					
ECOG 0	965	39	333	34	0.15
ECOG 1	370	15	133	13	
ECOG 2	169	7	82	8	
ECOG 3/4	50	2	31	3	
Unknown	938	38	413	42	
Renal function (mL/min/1.73m^2^)					
0–39	342	14	136	14	0.05
40–59	533	21	216	22	
60–79	647	26	253	26	
≥ 80	636	26	251	25	
Unknown	334	13	136	14	
Clinical stage					
T2, N0, M0	1,803	72	637	64	0.19
T3, N0, M0	552	22	259	26	
T4a, N0, M0	137	5	96	10	
Histology					
Urothelial carcinoma	2,334	94	893	90	0.22
Squamous cell carcinoma	22	1	44	4	
Adenocarcinoma	7	0	2	0	
Neuro-endocrine carcinoma	96	4	29	3	
Undifferentiated carcinoma	4	0	4	0	
Other/undetermined	29	1	20	2	

*IQR*  interquartile range, *CCI* Charlson Comorbidity Index, *ECOG* Eastern Cooperative Oncology Group

For the entire cohort of MIBC patients, women less often received treatment with curative intent (55% vs 59%, standardized difference: 0.08). In addition, within all strata of T-stage, the initial treatment provided was more often with curative intent for men. While for cT2 and cT3 tumors, the difference was small (standardized differences 0.03 and 0.13) between men and women (59% vs 56% and 61% vs 57%), for cT4a tumors only 38% of the women received curative treatment compared to 50% of the men (Table 2), corresponding to a standardized difference of 0.25. The difference in type of treatment administered to men and women with cT4a disease differed the most (standardized difference of 0.37), with lower proportions of women receiving radical cystectomy, trimodal therapy and systemic therapy, and a slightly higher proportion receiving radiotherapy.

**Table 2:** Initial treatment provided for all muscle-invasive, non-advanced bladder cancer patients in the Netherlands diagnosed in 2018–2020, by clinical T-stage and sex

	Men		Women		Standardized differences
	*N*	%	*N*	%	
All stages	2492	100	992	100	
Treatment					
RC + NAT	330	13	151	15	0.13
RC−NAT	804	32	286	29	
Trimodal therapy	331	13	106	11	
Radiotherapy	484	19	207	21	
Systemic treatment	52	2	24	2	
Other/none*	491	20	218	22	
Curative treatment**					
Yes (RC/TMT)	1465	59	543	55	0.08
No	1027	41	449	45	
cT2, N0, M0	1803	100	637	100	
Treatment					
RC + NAT	182	10	75	12	0.12
RC−NAT	622	34	201	32	
Trimodal therapy	254	14	83	13	
Radiotherapy	367	20	140	22	
Systemic treatment	20	1	8	1	
Other/none*	358	20	130	20	
Curative treatment**					
Yes (RC/TMT)	1058	59	359	56	0.03
No	745	41	278	44	
cT3, N0, M0	552	100	259	100	
Treatment					
RC + NAT	115	21	59	23	0.15
RC−NAT	156	28	68	26	
Trimodal therapy	67	12	21	8	
Radiotherapy	99	18	52	20	
Systemic treatment	20	4	9	3	
Other/none*	95	17	50	19	
Curative treatment**					
Yes (RC/TMT)	338	61	148	57	0.13
No	214	39	111	43	
cT4a, N0, M0	137	100	96	100	
Treatment					
RC + NAT	33	24	17	18	0.37
RC−NAT	26	19	17	18	
Trimodal therapy	10	7	2	2	
Radiotherapy	18	13	15	16	
Systemic treatment	12	9	7	7	
Other/none*	38	28	38	40	
Curative treatment**					
Yes (RC/TMT)	69	50	36	38	0.25
No	68	50	60	63	

*RC*  radical cystectomy; *NAT*  neoadjuvant treatment, including chemotherapy and immunotherapy; *TMT*  trimodal therapy

^*^Other/none includes local treatments such as transurethral resection of the tumor, bladder instillations and partial cystectomies

**Curative treatment includes radical cystectomy with or without neoadjuvant treatment and trimodality treatment

The type of treatment administered per cT stratum was assessed by hospital type. The difference of undergoing curative treatment or no tumor-directed treatment between men and women was strongest among patients diagnosed in general hospitals, and weaker or absent in university hospitals (Supplementary Table 1).

Aspects of RC treatment were assessed separately, as the most common treatment modality. Among the patients receiving RC, women more often received neoadjuvant treatment for cT2 and cT3 disease, but less often for cT4a disease. In the vast majority of the cases, neoadjuvant treatment was cisplatin-based chemotherapy. The surgical technique was more often open for women (53% vs 50%) and more often robot-assisted for men (45% vs 42%). The urinary diversion was more often continent for men (6 vs 3%) (Table 3).

**Table 3:** Aspects of radical cystectomies for treatment for muscle-invasive, non-advanced bladder cancer by sex

	Men		Women		Standardized differences
	*N*	%	*N*	%	
All	1134	100	437	100	
Neoadjuvant treatment	330	29	151	35	
Among cT2 patients					
Yes	182	23	75	27	0.11
No	622	77	201	73	
Among cT3 patients					
Yes	115	42	59	46	0.08
No	156	58	68	54	
Among cT4a patients					
Yes	33	56	17	50	0.12
No	26	44	17	50	
Type (all stages)					
Cisplatin	280	85	127	84	0.15
Carboplatin	22	7	12	8	
Cis/carbo cross-over	11	3	8	5	
Other, incl. immunotherapy	17	5	4	3	
Surgical approach					
Surgery technique					
Open	571	50	233	53	0.06
Scopic (including robot-assisted)	543	48	194	44	
Other/unknown	20	2	10	2	
Type of cystectomy					
Sexually preserving	21	2	13	3	0.06
Standard	1102	97	418	96	
Other/unknown	11	1	6	1	
Urinary diversion					
Continent	65	6	11	3	0.15
Incontinent	1055	93	418	96	
Unknown diversion	14	1	8	2	

**Fig. 1:**
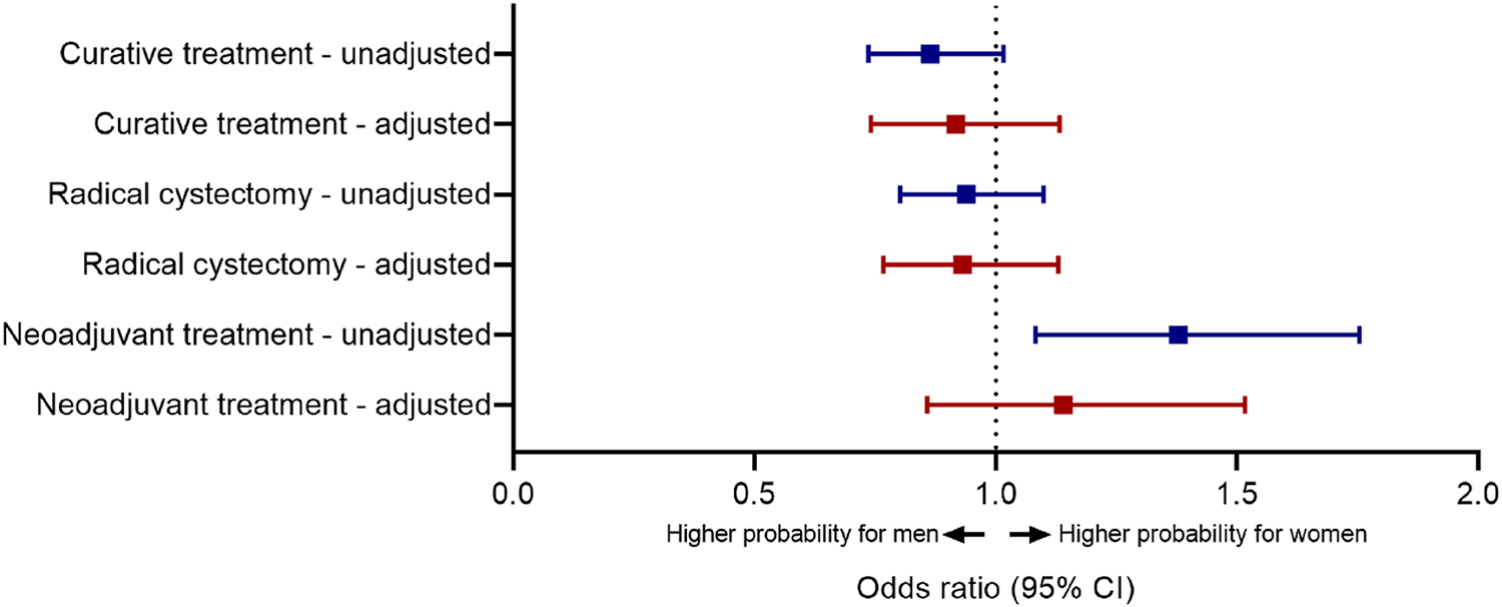
Univariable and multivariable analysis of association between sex and treatment among men and women with muscle-invasive bladder cancer. Adjusted odds ratios are adjusted for age, age-adjusted Charlson comorbidity index, performance status, renal function, cT-stage, and histology and hospital type

Multivariable logistic regression showed that the probability of undergoing treatment with curative intent, adjusted for age, age-adjusted Charlson comorbidity index, performance status, renal function, cT-stage, histology and hospital type was slightly lower for women, but non-significant (OR 0.92; 95%-CI 0.75–1.13) (Fig. 1). The same was found for radical cystectomy specifically (OR 0.95; 95%-CI 0.78–1.14). Among RC-treated patients, women had slightly higher but non-significant probability of undergoing neoadjuvant treatment after adjustment for pre-treatment factors (OR 1.14; 95%-CI 0.86–1.51).

## Discussion

These analyses among a contemporary nationwide population-based cohort highlights several important differences in treatment patterns among men and women with MIBC. Overall and within strata of clinical stage, women with MIBC were less often treated with curative intent, which was most notable in higher T-stages. In some patients, neoadjuvant treatment with RC may have been planned, but RC was canceled due to change in perceived benefit or feasibility after neoadjuvant treatment was given. This was the case in 26 patients (7 female), so the impact of this on sex differences should be negligible. In addition, physicians’ preferences and patients’ choices may have contributed to the observed differences in treatment patterns, but data for this were not available to determine the extent.

Radical cystectomy comprises the main curative treatment option among MIBC patients, with some aspects of the procedure found to vary between men and women. Women were more likely to be treated with neoadjuvant therapy prior to RC among cT2/3 patients, but less likely among cT4a patients. In addition, among patients undergoing RC, men more often had continent urinary diversions. When considering the type of hospital in which patients were diagnosed, general hospitals seemed to be accounting for more differences in treatment patterns between men and women compared to non-university teaching hospitals and university hospitals.

After taking into account all other pre-treatment factors that may weigh in on treatment decisions in addition to the patient’s sex, the patient’s sex was not significantly associated with receiving curative treatment in general, receiving RC specifically, or receiving neoadjuvant treatment when undergoing RC. Nonetheless, adjusted odds ratios were indicative of women having lower probability of receiving curative treatment and radical cystectomy specifically, and higher probability to receive NAC when treated with RC then men. This suggests that other pre-treatment factors for which the odds ratios were adjusted only account for part of the association of sex with treatment. A SEER-based study in the U.S. found that women were more likely to undergo radical cystectomy, both with and without adjustment for other factors, as compared to any alternative (including only TUR) [23]. In the current cohort, this was not the case, but patient populations seem to differ in relevant aspects including age and nodal status from the SEER cohort. In another US based cohort study using the National Cancer Database that distinguished between different alternatives to RC including non-curative options, the proportion of curatively treated patients was indeed also higher among male patients than female patients [24]. Both these studies included patients back to 2004, when TMT was not often used.

The observed sex disparity in treatment allocation is particularly striking in cT4a tumors, with 50% of the men undergoing RC compared to only 38% of the women, although this was based on limited numbers of patients. The staging of cT4a tumors takes into account gender-specific anatomy, with T4 disease defining tumor extension into the prostatic stroma in men and extension into the vagina or uterus in women [25]. In a single-center analysis of 176 patient with pT4a disease, the 1-year cancer-specific mortality rate was higher for invasion of the vagina (71%) or uterus (65%) compared to the invasion of the prostate only (24%) or with concomitant invasion of the seminal vesicles (50%) [26]. With regard to pT4a stage, a significantly lower 5-year cancer-specific survival was found in women (15%) compared to men (35%) [27] and female sex was an adverse prognostic factor for cancer-specific mortality (CSM) [28]. It is, therefore, conceivable that in light of these data and the anatomic differences, RC is considered a less appropriate treatment approach in women than in men with cT4a tumors and is, therefore, less often applied to women. With the TMT not considered an appropriate treatment option for higher T-stages, this could explain (part of) the discrepancy in proportion of curatively treated women with T4a tumors.

The following limitations of this study should be considered. At the individual patient level, several factors that can influence treatment options and choice, including patient preference, were not represented in our data. In addition, although it is likely that higher proportions of treatments that have been established to yield inferior outcomes among women are also associated with worse survival, this has not been directly demonstrated by the current study.

In the era of precision oncology, the selection of patient who might benefit from curative treatment approaches needs to be improved. The sex of the patients is one of the most obvious and important factors affecting the efficacy and toxicity of cytotoxic treatments, with women in general having higher response rates as well as toxicity rates for a variety of anticancer treatments [29]. Indeed, in a recent meta-analysis, women benefited more from neoadjuvant chemotherapy prior to RC, improved disease recurrence and CSM rates compared to men [30]. These data indicate that increasing rates of curative treatment approaches for women could improve the outcome of patients.

To conclude, considerable differences in treatment patterns between men and women with MIBC exist. A more considerate role of the patient’s sex in treatment decisions could help decrease these differences and might mitigate disparities in outcomes.

## Supplementary Information

Below is the link to the electronic supplementary material.

Supplementary file1.
