# Supplementary material for: Sex differences in treatment patterns for non-advanced muscle-invasive bladder cancer: a descriptive analysis of 3484 patients of the Netherlands Cancer Registry
Source: World J Urol. 2022 Jul 1;40(9):2275–81. doi: 10.1007/s00345-022-04080-6 (PMC9427875; doi:10.1007/s00345-022-04080-6)
Supplement: Supplementary file 1 — Supplementary file1. [file 345_2022_4080_MOESM1_ESM.docx]

# Supplementary Table 1: Initial treatment for muscle-invasive, non-advanced bladder cancer by type of hospital

|  | **Type of diagnosis hospital** | | | | | | | | | | | |
| --- | --- | --- | --- | --- | --- | --- | --- | --- | --- | --- | --- | --- |
|  | **General  hospital** | | | | **Teaching  hospital** | | | | **University  hospital** | | | |
|  | **Men** | | **Women** | | **Men** | | **Women** | | **Men** | | **Women** | |
|  | N | % | N | % | N | % | N | % | N | % | N | % |
| **cT2, N0, M0 (row%)** | **474** | **19%** | **179** | **7%** | **986** | **40%** | **347** | **14%** | **341** | **14%** | **111** | **5%** |
| RC + NAT | 26 | 5% | 20 | 11% | 112 | 11% | 40 | 12% | 44 | 13% | 15 | 14% |
| RC – NAT | 102 | 22% | 32 | 18% | 373 | 38% | 126 | 36% | 146 | 43% | 43 | 39% |
| Trimodal therapy | 69 | 15% | 22 | 12% | 98 | 10% | 33 | 10% | 87 | 26% | 28 | 25% |
| Radiotherapy | 136 | 29% | 49 | 27% | 189 | 19% | 75 | 22% | 42 | 12% | 16 | 14% |
| Systemic treatment | 7 | 1% | 2 | 1% | 12 | 1% | 4 | 1% | 1 | 0% | 2 | 2% |
| Other/none* | 134 | 28% | 54 | 30% | 202 | 20% | 69 | 20% | 21 | 6% | 7 | 6% |
| **cT3, N0, M0 (row%)** | **127** | **16%** | **59** | **7%** | **277** | **34%** | **130** | **16%** | **148** | **18%** | **70** | **9%** |
| RC + NAT | 19 | 15% | 6 | 10% | 59 | 21% | 24 | 18% | 37 | 25% | 29 | 41% |
| RC – NAT | 19 | 15% | 8 | 14% | 85 | 31% | 37 | 28% | 52 | 35% | 23 | 33% |
| Trimodal therapy | 11 | 9% | 4 | 7% | 22 | 8% | 8 | 6% | 34 | 23% | 9 | 13% |
| Radiotherapy | 44 | 35% | 21 | 36% | 38 | 14% | 27 | 21% | 17 | 11% | 4 | 6% |
| Systemic treatment | 5 | 4% | 1 | 2% | 11 | 4% | 6 | 5% | 4 | 3% | 2 | 3% |
| Other/none* | 29 | 23% | 19 | 32% | 62 | 22% | 28 | 22% | 4 | 3% | 3 | 4% |
| **cT4a, N0, M0 (row%)** | **35** | **15%** | **27** | **12%** | **61** | **26%** | **53** | **23%** | **41** | **17%** | **16** | **7%** |
| RC + NAT | 2 | 6% | 1 | 4% | 14 | 23% | 8 | 15% | 17 | 41% | 8 | 50% |
| RC – NAT | 3 | 9% | 2 | 7% | 11 | 18% | 13 | 25% | 12 | 29% | 2 | 13% |
| Trimodal therapy | 4 | 11% | 1 | 4% | 2 | 3% | 1 | 2% | 4 | 10% | - | - |
| Radiotherapy | 7 | 20% | 4 | 15% | 7 | 11% | 9 | 17% | 4 | 10% | 2 | 13% |
| Systemic treatment | 6 | 17% | 3 | 11% | 5 | 8% | 3 | 6% | 1 | 2% | 1 | 6% |
| Other/none* | 13 | 37% | 16 | 59% | 22 | 36% | 19 | 36% | 3 | 7% | 3 | 19% |

** Two patients are not in this table because they were diagnosed in a hospital outside of the Netherlands.*
